# The Effect of Brewing Process Parameters on Antioxidant Activity and Caffeine Content in Infusions of Roasted and Unroasted Arabica Coffee Beans Originated from Different Countries

**DOI:** 10.3390/molecules26123681

**Published:** 2021-06-16

**Authors:** Anna Muzykiewicz-Szymańska, Anna Nowak, Daria Wira, Adam Klimowicz

**Affiliations:** Chair and Department of Cosmetic and Pharmaceutical Chemistry, Pomeranian Medical University in Szczecin, Powstańców Wielkopolskich Ave. 72, 70-111 Szczecin, Poland; anna.nowak@pum.edu.pl (A.N.); daria.wira@pum.edu.pl (D.W.); adam.klimowicz@pum.edu.pl (A.K.)

**Keywords:** coffee Arabica, roasting process, brewing methods, antioxidant activity, polyphenols, flavonoids, caffeine, pH of infusions, tannins

## Abstract

Coffee is one of the most often consumed beverages almost all over the world. The multiplicity of beans, as well as the methods and parameters used to brew, encourages the optimization of the brewing process. The study aimed to analyze the effect of roasting beans, the brewing technique, and its parameters (time and water temperature) on antioxidant activity (determined using several in vitro methods), total polyphenols, flavonoids, and caffeine content. The infusions of unroasted and roasted Arabica beans from Brazil, Colombia, India, Peru, and Rwanda were analyzed. In general, infusions prepared from roasted beans had higher antioxidant activity and the content of above-mentioned compounds. The hot brew method was used to obtain infusions with a higher antioxidant activity, while the cold brew with higher caffeine content. The phenolic compound content in infusions prepared using both techniques depended on the roasting process. Moreover, the bean’s origin, roasting process, and brewing technique had a significant effect on the tested properties, in contrary to brewing time and water temperature (below and above 90 °C), which had less impact. The results confirm the importance of coffee brewing optimization.

## 1. Introduction

Coffee trees belong to the constantly green trees and shrubs of the Rubiaceae family [1]. The optimal conditions for the growth of different coffee varieties occur in so-called the Coffee Belt located in the intertropical areas [2]. Ninety-five percent of global production of Coffea Arabica and Coffea Robusta are used for consumption, especially Coffea Arabica due to its delicate and less bitter taste [3]. An annual production of green coffee beans is about nine million tons. Even though Ethiopia is the cradle of Coffea Arabica [4], Brazil is the greatest world coffee exporter [1]. Other major suppliers of coffee beans are South American countries (Colombia and Peru) as well as African and Asian, such as Rwanda, above-mentioned Ethiopia, and Vietnam, Indonesia, and India [4].

After harvesting, the coffee beans are selected, shelled, and roasted at 200–250 °C [5]. The desired aroma and flavor of coffee are related to the volatile and non-volatile compounds. Their content is affected by the selection of appropriate roasting parameters and the subsequent Maillard reaction, which additionally leads to a change of beans color as a result of the production of melanoidins [6]. There are many techniques of coffee brewing. Generally, they can be divided into high- and low-temperature processes. The most popular methods of hot coffee brewing are the Turkish technique, French press, Aeropress, espresso, and simple infusion method [7,8]. Turkish coffee preparation involves boiling a mixture of coffee with water. The brew cannot be filtered [8]. A simple infusion based on pouring the ground grains with hot water (~85–95 °C) and macerated for about 5 min [7,9]. During the preparation of espresso, the roast ground coffee is briefly pressurizing with hot water using a percolator to obtain a small concentrated cup of coffee. The pressure is one of the most important parameters of the coffee brewing process [10]. Aeropress created in 2005 by Adler is a device that uses pressure, overflow, and French press techniques. Hot water and the short brewing time (~2 min) are used to prepare infusions [7,9]. Methods of cold coffee extraction include direct and indirect immersion methods, dripping, and the French press [10]. To prepare cold brew coffee using the direct method, water at room temperature is applied, as in the cold drip case. Then, the ground coffee beans are immersed in a proper amount of water for at least 6–8 up to 24 h followed by filtration [11,12]. In the cold drip method, water at a temperature of 20–25 °C is slowly dripped onto coffee powder placed in the filter to obtain a relatively intensive coffee extract in the beaker. For this purpose, one drop of water is added usually every 5 or 10 s. Recently, a French press method, the so-called plunger pot, has gained interest. This technique uses the pot with the plunger, to which hot or cold water is poured inside [7,10]. It allows to get fresh unfiltered infusion immediately. French press coffee maker enables to depress the plunger to separate coarsely ground coffee beans from the liquid [13].

Many researchers confirmed the health-promoting properties of consuming coffee brew. The most important benefits are summarized in Table 1. In addition to the taste, coffee beans are a valuable source of bioactive compounds, whose profile depends on coffee species, thermal processing, cultivating conditions, and harvesting time [11,14]. Due to the content of antioxidants, as well as caffeine, the brews of coffee belong to the functional food sector [14]. The caffeine content in seeds ranges from 0.3% to 2.5% and is twice as high in Coffee Robusta as in Coffee Arabica [3,5]. Ground coffee brews are also rich in phenolic compounds, such as chlorogenic acid, hydroxycinnamic acids, and their derivatives such as caffeic and ferulic acids and alkaloids (mainly caffeine and small amounts of theobromine and theophylline), diterpenoid alcohols (cafestol and kahweol), carbohydrates, lipids, and volatile and heterocyclic compounds [5,15].

There are many reports on the coffee brewing process, but most of them are focused on the assessment of selected parameters, such as the impact of the intensity of the roasting process, the thickness of the grinding, or the method of brewing. The present study aimed to evaluate several parameters of the coffee brewing process, such as the method of making infusions (cold and hot brew), water temperature (cold, below or above 90 °C), and brewing time (9 and 24 h as well as 4 and 10 min). The details of the various brewing methods are presented in Table 2. For this purpose, unroasted and roasted Arabica coffee beans, imported from various countries (Brazil, Colombia, India, Peru, and Rwanda), belonging to the world’s largest suppliers of this raw material, were analyzed. The influence of these factors on the in vitro antioxidant activity of infusions as well as on the content of polyphenols, flavonoids, and caffeine was analyzed. The results show that the bean’s origin, roasting process, and brewing technique had a significant effect on the tested properties. The factors such as the difference in brewing time and water temperature (below and above 90 °C) had less impact. The obtained results could extend our knowledge on the brewing process of the most frequently consumed beverage in the world to verify parameters affected the content of health-promoting biologically active compounds, especially antioxidants.

## 2. Results and Discussion

### 2.1. In Vitro Antioxidant Activity of Coffee Infusion

**Figure 1:**
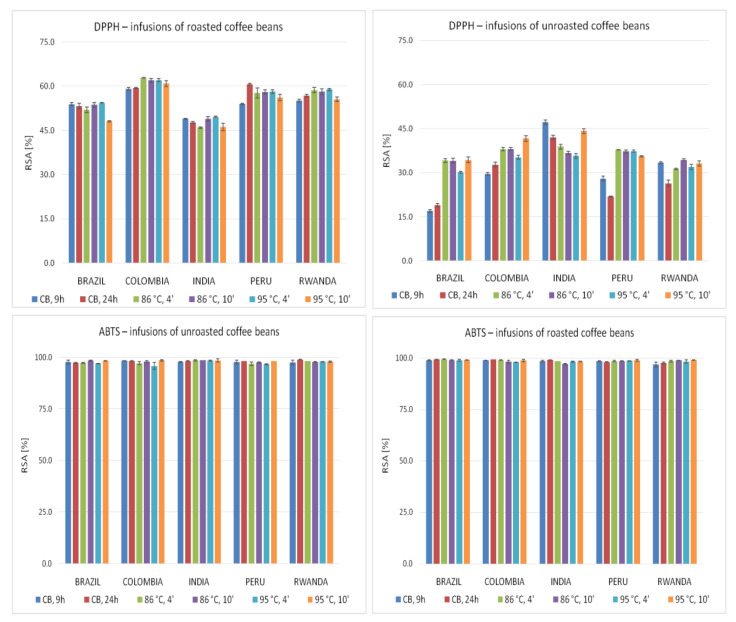
Radical scavenging activity (RSA [%]) of coffee infusions evaluated using DPPH and ABTS methods. Vertical lines represent standard deviation (SD). Details regarding brewing methods are summarized in Table 2.

**Figure 2:**
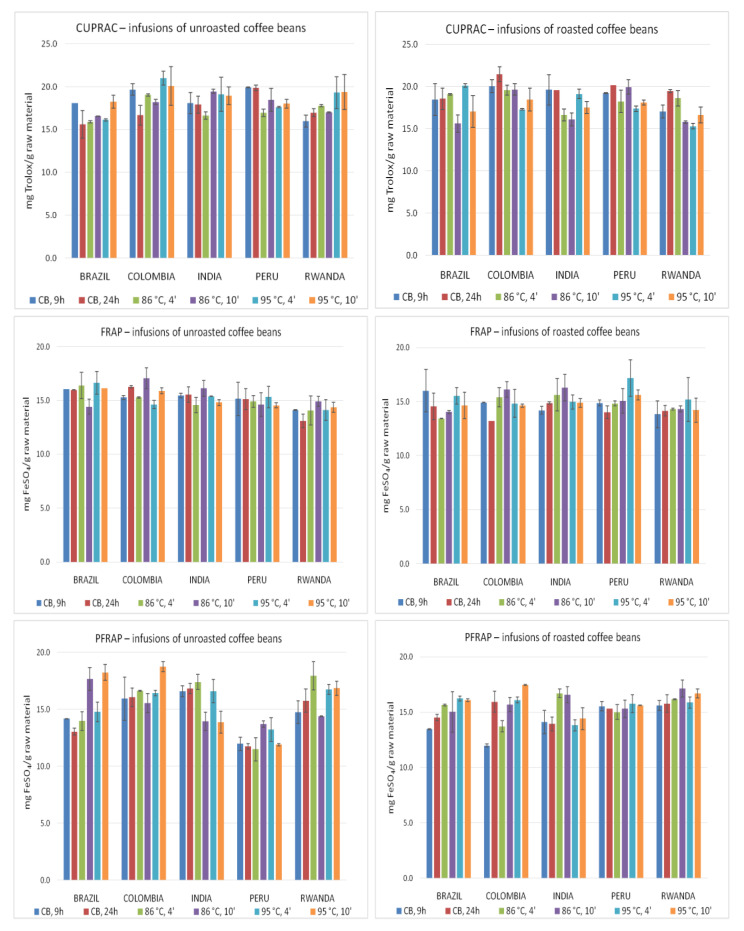
The ability of coffee infusions to reduce cupric and ferric ions determined by CUPRAC, FRAP, and PFRAP methods. Vertical lines represent standard deviation (SD). Details regarding brewing methods are summarized in Table 2.

Figure 1 presents free radical scavenging activities (RSA [%]) of infusions prepared using various brewing methods from unroasted (green) and roasted (brown) coffee beans from different countries, assessed by the DPPH and ABTS techniques. The brew’s abilities to reduce ferric ions evaluated by the FRAP and PFRAP methods as well as to reduce cupric ions determined with CUPRAC method are presented in Figure 2.

The infusions prepared from roasted beans were characterized by a higher antioxidant potential expressed as RSA [%] evaluated by DPPH technique (Figure 1), as compared to infusions from unroasted beans. The highest activity evaluated using DPPH method was found for the Colombian roasted coffee beans infusion (86 °C, 4 min), whereas the lowest for the Brazilian green coffee beans infusion brewed using the cold brew method for 9 h. The highest RSA [%] in the roasted coffee beans infusions group prepared using different brewing methods was most often observed for extracts from Colombian beans, whereas the lowest was for Indian bean brews. In the case of unroasted beans, the highest activity of infusions was found for Indian coffee, whereas the lowest was for infusions prepared from Brazil and Rwanda coffee beans. Prolongation of roasted beans brewing time generally increased the activity of cold brew infusions but reduced the activity of infusions brewed at a temperature above 90 °C. In the case of unroasted beans, the increase of activity due to the extension of the brewing time was observed only for coffee brewed at a temperature above 90 °C. All the results obtained by DPPH method could suggest that the hot brew method led to obtain infusions with higher antioxidant activity.

The infusions analyzed using ABTS method (Figure 1) were characterized by very high activities, ranging from 95.76% RSA (green beans from Colombia, brewed at 95 °C for 4 min) to 99.43% RSA (infusion of roasted coffee from Brazil—86 °C, 4 min). As a rule, the roasted coffee bean infusions were characterized by a higher antioxidant potential than analogously prepared extracts from unroasted beans. The highest activities of the infusions prepared from roasted coffee beans were observed most often for Brazilian beans, while in the group of extracts from green coffee beans, for the brews from Indian and Colombian beans. In the group of unroasted beans extracts, it was also found that infusions of Peru beans are frequently characterized by slightly lower activity assessed by ABTS method. However, prolongation of brewing time led to enhance of the antioxidant activity, especially for extracts prepared from unroasted beans. This tendency was also found for infusions prepared with the cold brew method and at a temperature above 90 °C. Therefore, infusions prepared using the cold and hot brew techniques showed a very high free radical scavenging potential evaluated using the ABTS technique; however, it is rather difficult to unequivocally evaluate the effect of brewing temperature on antioxidant activity of brews.

The reduction potential of the infusions was evaluated using the FRAP, PFRAP, and the CUPRAC methods (Figure 2). The highest activity determined by the FRAP method was found for roasted beans from Peru (95 °C, 4 min), while the lowest for the unroasted beans infusion from Rwanda beans (CB, 24 h). Moreover, it was found that activity of extracts from unroasted beans was often higher as compared to infusions prepared from roasted beans. This tendency was especially observed for infusions prepared using the cold brew method. In the case of roasted beans, the highest activity was often observed for coffee beans from India and Peru, and the lowest for beans from Colombia and Rwanda. Moreover, the highest activity evaluated by FRAP method was found for unroasted Brazil and Colombia beans, whereas the lowest for Rwanda coffee. Evaluation of the brewing time effect of roasted and unroasted beans showed that in the case of the cold method and during application water of above 90 °C, a shorter brewing time seemed to be more effective. In the case of the hot brew method using water at a temperature of ~85 °C, brewing for 10 min seems to be more effective. The impact of brewing method on the infusion’s potential showed that the hot brewing technique led to obtain infusions with a higher reduction activity.

The highest reduction activity of iron ions assessed by the PFRAP method (Figure 2) was observed for unroasted Colombian coffee beans infusion (95 °C, 10 min), whereas the lowest for unroasted beans from Peru (CB, 24 h). Based on the analysis of the results obtained for roasted and unroasted coffee beans, it is rather difficult to clearly define the group showed higher activity. However, the highest potential for roasted beans was found for infusions brewed at 86 °C, whereas for unroasted beans at 95 °C. Moreover, in the group of unroasted and roasted beans infusions, the highest activities were obtained most often for extracts from Rwanda beans. Additionally, also roasted Indian coffee beans brews were highly active. The lowest potential showed the infusions prepared from Peru coffee, both roasted and unroasted. In contrary to roasted, unroasted Indian coffee bean infusion had generally quite low antioxidant potential. The analysis of brewing time has established no beneficial effect of its prolongation on the tested potential of the extracts. The results suggested that in the case of hot brew (brown beans), 10 min process is generally more effective, while in the case of unroasted beans (85 °C)—4 min. Similar to the FRAP method, the analysis of the impact of the brewing method showed that hot coffee brewing techniques led to infusions with a greater reduction in the activity of ferric ions evaluated using PFRAP technique.

Application of CUPRAC method (Figure 2) to determine cupric ion reduction capacity suggest that Colombian roasted coffee extract (CB, 24 h) was characterized by the highest activity, whereas the lowest was found for the infusion prepared using the same method from Colombian unroasted beans. Comparison of the activities of roasted and unroasted coffee bean infusions leads to the conclusion that after application either the cold brewing method or 86 °C (4 min) roasted coffee bean infusions are more active, in contrary to the other brewing methods, where the green bean extracts were characterized by higher potential. Moreover, the highest ability to reduce cupric ions was found most often for Colombian coffee extracts (both roasted and unroasted), while the lowest for Rwanda roasted and Brazil unroasted coffee beans. Shorter brewing time was more effective to prepare infusions from brown and green beans using water below 90 °C and the cold brew method for unroasted beans. In the other brewing methods, prolongation of brewing time up to 24 h in cold method and up to 10 min in hot method seems to be more efficient.

The analysis of the effect of roasting coffee beans on the antioxidant activity of infusions showed that as a rule antioxidant potential of roasted coffee beans was higher, as compared to unroasted beans. Priftis et al. [21] evaluated the antioxidant activity of 13 varieties of coffee (roasted and unroasted) and confirmed that both types of coffee showed the high ability to scavenge free radicals. The impact of roasting on the activity of the obtained infusions depended on the variety of beans. This phenomenon may be related to the different chemical composition of beans. Moreover, the burning time also influenced the antioxidant activity of the obtained extracts. Dybkowska et al. [22] analyzed the antioxidant properties of coffee brews prepared from beans cultivated in Brazil, Ethiopia, Colombia, and India. The beans with various roasting degrees were analyzed—light, medium, and dark. They noticed that the process of roasting coffee beans increased the antioxidant potential of the obtained infusions. This phenomenon is directly related to the formation of melanoidins as a result of the Maillard reaction in coffee beans upon roasting. Melanoidins influence the antioxidant activity of coffee and its sensory properties. Their content in coffee depends, among others, on the intensity of the roasting process—the higher the roasting temperature, the higher content of these compounds, but lower their molecular weight. Ribeiro et al. [23] confirmed a higher content of melanoidins in roasted coffee bean infusions than in unroasted. In their study, higher antioxidant activity evaluated using FRAP and ABTS methods was found for roasted bean infusions as compared with infusions prepared from green coffee beans. Hečimović et al. [24] also confirmed our observations, that both factors—coffee variety and the roasting degree—affect the antioxidant activity. In their study, similarly to ours, infusions prepared from unroasted beans showed a lower potential than extracts from roasted beans.

The country of the coffee bean’s cultivation could also affect the antioxidant activity of infusions. High antioxidant potential was found quite often for coffee from Colombia and India, while infusions from beans from Peru and Rwanda were characterized by lower activity. Oszmiański et al. [25,26], based on the apple analysis, concluded that the factors such as year of harvest, growth period, storage conditions, geographic location and genetic variation, the effect of the region, agricultural practices, and cultivation method could have an impact on the plant biochemical profile including antioxidant potential. It is assumed that the roasting degree of beans could have a significant impact, apart from the individual composition of particular varieties. The beans from Colombia and India were burned medium-light to medium. Coffee from Peru was the most roasted of all the tested varieties, in contrary to the least roasted from Rwanda. The observations are confirmed by the suggestion of Priftis et al. [21] that the roasting degree of coffee beans should be optimized for high antioxidant activity. Dybkowska et al. [22] also suggest that for nutritional reasons, consumption of coffee with a short or medium degree of burnout is the most beneficial for human health.

The analysis of the brewing method suggests that the hot technique could have a positive effect to obtain infusions with a higher antioxidant potential than the cold method. Rao and Fuller [27] showed that antioxidant activity of hot-brewed infusions was higher as compared to cold-brewed infusions. They suggested that this phenomenon could be related to the hot water extraction of additional bioactive compounds, including those with antioxidant potential. It may be related to the higher content of caffeoylquinic and chlorogenic acid isomers in hot-brewed coffee than in cold-brewed infusions. Rao et al. [28] also suggested that hot water can more easily wet the oily surface of coffee beans, to enhance the efficiency of extraction of active compounds, including those with antioxidant potential. Coffee brewing using hot or cold water could affect the different solubilities of compounds with higher molecular weight, mainly melanoidins.

Temperature of the water to be used to prepare infusions (hot brew method) is another parameter to be taken into consideration. The antioxidant activity of infusions brewing with water at a temperature of above 90 °C was higher. There are many reports on the effect of the roasting degree or the method of brewing coffee on the antioxidant activity of infusions. However, there is less information on the influence of small differences in water temperature on the activity of the obtained infusions. Castiglioni et al. [29] investigated the effect of water temperature (70 °C, 90 °C, and cold) on the antioxidant activity of white and green tea from China and Malawi. They found a maximum efficiency using water at temperature of 90 °C and cold brewing method. Pramudya and Seo [30] studied the effect of the temperature of serving coffee (5 °C, 25 °C, 65 °C) on the sensor attributes and emotional responses of the coffee brews. The respondents most often described the taste of coffee served at 65 °C as “roasted flavor”, while the taste of the brew served at 5 °C as “pungent aroma”, “metallic flavor”, and “skunky flavor”. They also perceived similar observations when serving green tea at different temperatures. The authors found that the respondents valued more positive sensory features of infusions served at higher than at lower temperature.

However, it is difficult to clearly evaluate how prolongation of the brewing time could affect the antioxidant activity of the infusions. Górecki and Hallmann [31] compared the antioxidant activity of infusions brewing for 3 and 6 min. They obtained slightly higher results for infusions prepared during a 6 min process. However, these differences were not significant. Moreover, the authors also suggest that other factors, such as the roasting degree of beans, could affect the antioxidant potential of the infusions.

### 2.2. Total Polyphenols and Flavonoids Content

**Figure 3:**
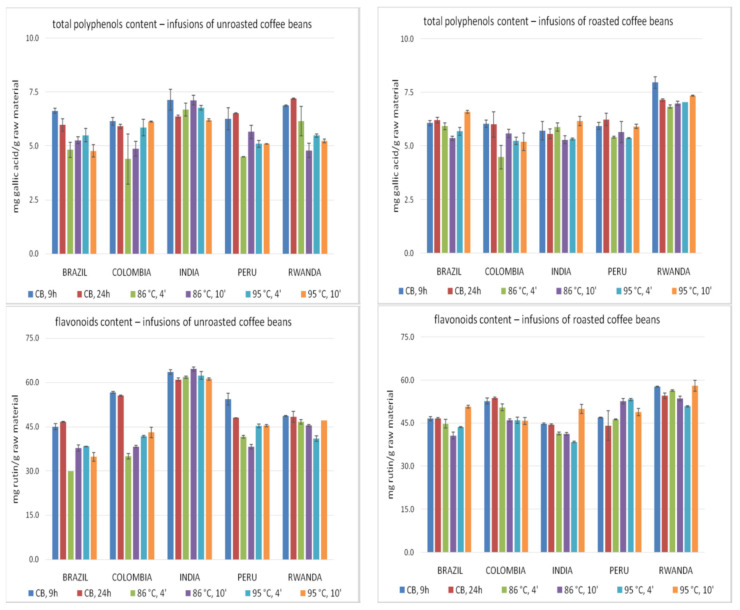
The total polyphenols and flavonoids content in coffee infusions. Vertical lines represent standard deviation (SD). Details regarding brewing methods are summarized in Table 2.

The total polyphenols and flavonoids content in the studied infusions are presented in Figure 3. The highest polyphenols content was found in the Rwanda roasted coffee beans infusion, brewed using the cold brew method for 9 h, whereas the lowest in unroasted Colombian coffee extract prepared during a 4-min brewing (86 °C). Generally, a higher content of polyphenols was found in roasted coffee bean infusions, particularly in those prepared using hot water. The cold brewing method was effective to obtain infusions with high content of polyphenols from unroasted coffee beans. In brews from roasted beans, the highest polyphenol content was found in the Rwanda coffee extracts, while the lowest in Colombian and Indian infusions. In unroasted bean infusions, the highest concentration of polyphenols was found most often in Indian coffee bean brews, while the lowest in Colombian. In the case of roasted beans, the extension of brewing time to 10 min in the hot brewing method usually increased the polyphenols content. However, no similar relationships were observed in the case of green coffee. The obtained results suggest that cold brewing increases the concentration of total polyphenols in infusions, as compared to traditional hot brewing.

The highest content of flavonoids (Figure 3) was found in the unroasted Indian coffee infusion (85 °C, 10 min), while the lowest in unroasted Brazilian coffee (85 °C, 4 min). In the group of infusions prepared with hot water, a higher content of flavonoids was found in extracts from roasted beans, while in the cold brew method, from unroasted beans. In unroasted bean infusions, the brews prepared from Indian coffee showed the highest flavonoid content, while Brazil beans the lowest concentration of these group of compounds. Among infusions obtained from roasted beans, the highest content of flavonoids was found for samples obtained from Rwanda beans, whereas the lowest for Indian coffee infusions. It is difficult to clearly assess the effect of time extension on the content of flavonoids in the tested brews. In cold brewing, the infusions prepared during the 9-h process are generally characterized by a higher content of the flavonoids. A shorter brewing time also seems to be rather optimal for brewing roasted coffee with water below 90 °C. Extending the brewing time to 10 min seemed to be more effective in the case of roasted beans brewing with water at 95 °C and green coffee with water below 90 °C. The obtained results suggest that in most cases the cold brewing contributes to obtain infusions with a higher content of flavonoids than hot brewing. Similar results were observed for total polyphenols content.

Similar to the antioxidant activity, it was found that roasted bean infusions contained more polyphenols, including flavonoids, than unroasted. The highest content of these compounds was found in Rwanda coffee characterized by the lowest roasting degree. Dybkowska et al. [22] showed a decrease in the content of polyphenols in 100% Arabica beans infusions and blend coffee of Arabica and Robusta beans because of the roasting process. The thermolabile nature of these compounds contributes to their degradation after prolonged exposure to high temperature. The authors also emphasize that the loss of polyphenols is unfavorable due to the health-promoting effect of these compounds on the human body. Król et al. [32] also indicate a decrease of polyphenols content in the brews because of the extension of the roasting beans process.

The brewing method could also affect the polyphenols and flavonoids content. Cold brewing infusions of unroasted beans lead to higher content of these compounds, while the hot brewing technique seems to be more effective for roasted beans. Fibrianto et al. [33] compared the effect of the hot and cold brewing method on the polyphenols content in infusions prepared from roasted Arabica beans. Similar to our results, they also found a higher content of these compounds in extracts obtained using hot water.

The evaluation of the effect of water temperature in the hot brewing method showed that a higher content of polyphenols and flavonoids was observed in slightly more infusions prepared with water at 95 °C rather than at 86 °C. Merecz et al. [34] investigated the effect of the brewing method (hot water, percolator, and coffee machine) on the content of polyphenols and flavonoids in brews. In their study, the roasted and unroasted Arabica and Robusta beans were evaluated. However, their results cannot clearly confirm the influence of the brewing method on the content of these compounds in infusions. The concentration of polyphenols and flavonoids depended on the type of coffee (species, roasted/unroasted, and country of the bean’s origins), as well as on the number of the brewings (1 to 3). In the case of roasted beans, the most effective method seemed to be application of a percolator (flooding the ground beans with cold water and then placed over a heat source). The content of polyphenols and flavonoids in unroasted beans was lower than in roasted. In the case of green coffee, the content of these compounds was higher in infusions prepared in a percolator and using hot water as compared to the brews from a coffee machine. Results of our study also lead to the conclusion that the type of coffee used (degree of roasting, country of origin, brewing method) had an impact on the content of biologically active compounds, including polyphenols and flavonoids in contrary to the slight differences in temperature of the water used to prepare the infusions. Merecz et al. [34] also emphasized that factors such as storage method and the degree of grinding beans could affect the profile of biologically active compounds in coffee infusions.

Furthermore, infusion time could affect the polyphenols content. In our study, it was found that extending of the brewing time, both in the cold (up to 24 h) and hot methods (up to 10 min), generally had a positive effect on the content of these compounds. In contrary, this tendency was not observed for the content of flavonoids, because in some cases, shorter brewing time seemed to be more optimal. Slightly different observations were made by Górecki and Hallmann [31]. They found that extending brewing time from 3 to 6 min contributed to a slight increase of flavonoids content and to a slight decrease of total phenols in coffee brews. However, these authors analyzed only the extracts prepared using the hot brewing technique. On the other hand, Cordoba et al. [11] confirmed our observation: extending the brewing time from 14 to 22 h (cold brewing method) increased the polyphenol content in coffee infusions.

### 2.3. Caffeine Content in Coffee Infusion

**Figure 4:**
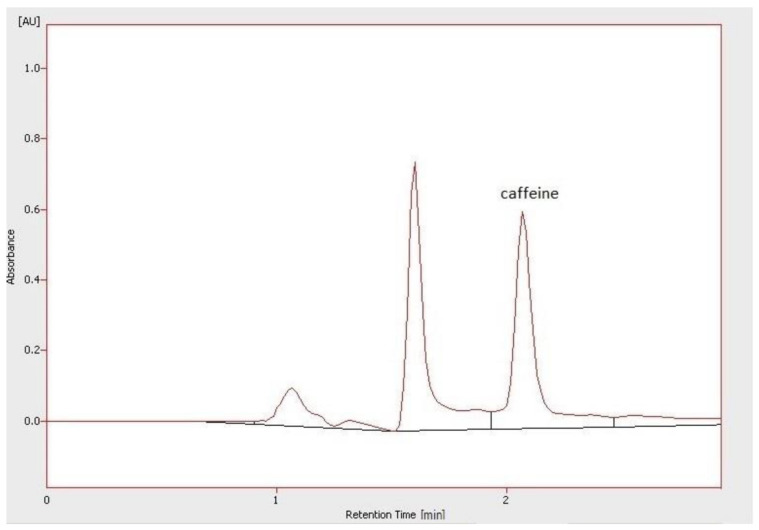
Chromatogram of the determination of caffeine in the infusion of unroasted beans from Peru (temperature of water—84–86 °C, brewing time—4 min).

The caffeine content in studied infusions is presented in Table 3, whereas the chromatogram presenting the analysis of the selected infusion (unroasted beans from Peru—86 °C, 4 min) in Figure 4. The highest caffeine content determined by HPLC method was found in the Indian roasted bean infusion (CB, 24 h), while the lowest in the Peru unroasted beans brews (95 °C, 4 min). The higher caffeine concentration was found rather in brown bean infusions than in green coffee beans. The cold method was more effective to prepare infusions with a higher caffeine content than the traditional hot brewing technique. However, infusions brewed with water at 95 °C for 4 min were characterized by the lowest caffeine content. The obtained results suggest that the relatively high content of caffeine (over 60 mg/100 mL in 5% (*w*/*w*) infusion) was found in brews prepared from Indian coffee. The lowest caffeine content was generally found in coffee from Peru and Rwanda, especially in brews obtained from unroasted beans.

Similar to the antioxidant activity as well as to the content of total polyphenols and flavonoids, higher caffeine concentration was usually found in roasted coffee infusions than in brews of green coffee beans. The highest caffeine content was observed in Indian coffee infusions, while the lowest concentrations were observed in coffee beans grown in Peru and Rwanda. Similar results, i.e., higher caffeine content in roasted than unroasted beans, were also obtained by Mubarak [14] and Motor and Beyen [35]. The influence of the roasting degree on the caffeine content is emphasized by Górecki and Hallmann [31]. They observed a significant decrease in caffeine content after long-term roasting of the beans. Moreover, they compared the content of this alkaloid in beans from conventional and organic crops. Conventional crops samples were characterized by a higher caffeine content, probably due to the use of nitrogen fertilizers that lead to increase the percentage of caffeine in coffee beans. Moreover, according to Gebeyehu and Bikila [36], the growing conditions of coffee trees could also modify the caffeine content in beans.

The evaluation of the effect of the brewing method showed that cold brew infusions usually are characterized by a high caffeine content. Moreover, it can be assumed that longer brewing time favor the preparation of infusions with a higher content of this alkaloid. Similar results were obtained by Fuller and Rao [37]. They found that cold-brew infusions were characterized by a higher caffeine content as compared to extracts prepared using the hot method. The authors suggest that the higher caffeine content in cold infusions may be caused, among others, by extending the brewing time. In their study, the time was extended from 6 min (hot brew) to even 24 h (cold brew). Such a procedure could increase the intragranular diffusion and decrease the concentration of extractable coffee compounds in the hot brew, as compared to the cold brew. Moreover, the extraction from the surface and near-surface matrix occurs more rapidly than the diffusion of compounds through the intragranular pore network to the grain surface. In another study, Rao et al. [28] compared, among others, the content of bioactive compounds in infusions prepared using cold and hot methods, from coffee beans with varying roasting degrees. They found that the roasting process led to several chemical and physical changes in the bean matrix. Depending on the water temperature using to prepare the infusion, the above-mentioned changes affected most likely the ability, speed, and permeation efficiency of the various compounds. In their study, the water temperature affected the caffeine content in the brews. As in the case of our research, cold brew infusions seemed to be more effective to obtain higher caffeine content than hot water infusions. The high caffeine content in cold drip infusions was also demonstrated by Córdoba et al. [38].

Moreover, the water temperature could affect the caffeine content. Infusions prepared using the hot brew method with water temperature not exceeding 90 °C were a little more often characterized by a higher content of this alkaloid. Caprioli et al. [39] analyzed the Arabica and Robusta beans infusions prepared in two espresso machines used different pressure and temperature to brew coffee—at a temperature of 88–92 °C at a pressure of 9 bar and a temperature of 92–98 °C at a pressure of 7 bar. The caffeine content in Arabica bean infusions was higher in brews prepared at a higher temperature and lower pressure, while for Robusta coffee at lower temperature and higher pressure. In another study, Caprioli et al. [40] confirmed our observations: it is rather difficult to find consistent comparative data in the literature on the influence of coffee brewing parameters on the concentration of biologically active compounds in brews. The reason for this may be as a rule the application of a non-standard brewing method, characterized by different parameters such as the coffee-water ratio, the degree of beans roasting as well as differences in the units of the presented results. These factors can make it difficult to compare the data from different studies.

The analysis of the impact of brewing time on the caffeine content suggests that for the tested coffee varieties in our study, a longer brewing time seems to be more optimal. Similar results were obtained by Fuller and Rao [37]. In their study, the extending of brewing time from 400 to 1440 min increased the caffeine content in coffee infusions. The influence of the brewing method on the content of biologically active compounds, including caffeine, is also mentioned by Zaguła et al. [41]. The authors analyzed the influence of the application of a variable magnetic field on the caffeine content in black and green tea infusions. They suggested that a magnetic field assisted extraction could enhance the effectiveness of extracting the active substances from tea leaves to the infusion. Moreover, the authors emphasized that properly selected techniques designed to facilitate water-based extraction, using ultrasounds, magnetic fields, or microwaves may lead to technological advancements in the extraction of bioactive compounds from plant material.

### 2.4. Tannins Content and pH of Coffee Infusions

In our study, all the analyzed infusions contained tannins. The tannins in roasted coffee beans infusions were also found by Choi and Koh [42]. Patay et al. [43] also confirmed the content of these compounds in ripe and unripe seeds and pericarp of coffee beans.

The pH of all tested infusions was slightly acidic (Table 4). The pH of the unroasted bean infusions ranged from 5.16 (beans from Rwanda, cold-brewed for 9 h) to 6.58 (beans from Peru, brewed for 4 min with water at 95 °C). The pH of roasted bean infusions was more acidic, from 4.99 (Rwanda coffee, cold-brewed for 9 h) to 5.71 (Indian beans, cold-brewed for 24 h). Fibrianto et al. [33] analyzed the pH of infusions depending on the degree of roasting beans (light, medium, and dark). As the degree of roasting increased, the pH of the infusions was more alkaline. Slightly roasted bean infusions were more acidic.

The analysis of the influence of the brewing method on the pH of the obtained infusions showed that in the case of unroasted beans, cold brewing leads to extracts with a lower pH, while the hot method, with a higher pH. The opposite relationship was noticed in the group of roasted bean infusions, as application of the hot-brew method generally led to more acidic infusions than with the cold-brew method. The exception was coffee from Rwanda (CB, 9 h) with the lowest pH. Rao and Fuller [27] compared the pH of infusions of roasted beans from different countries, prepared using the hot- and cold-brew methods. In most cases, cold infusions were characterized by a more alkaline reaction. These observations were confirmed in our study. Rao and Fuller [27] also noted that coffee vendors often suggested that infusions prepared using cold- and hot-brew methods were characterized by different taste profiles due to different acidity levels. Therefore, it is believed that consumption of cold-brewed coffee, due to its lower acidity, could cause fewer gastrointestinal symptoms, sometimes observed after consuming coffee infusions. Rao and Fuller [27] clearly distinguish the pH assessment of infusions and their total titratable acidity. pH refers to the concentration of aqueous hydrogen ions, providing a metric for the quantity of deprotonated acid molecules in a tested sample, whereas the total titratable acidity is a measure of all acidic protons in a sample, including non-dissociated protons. Based on the obtained results, authors concluded that coffee infusions prepared using cold and hot brewing technique are similar, taking into account the total concentration of deprotonated acid compounds; however, they differ in the concentration and possibly the complexity of protonated acids at the pH of extraction. No correlation between perceived acidity in the flavor of coffee brews and pH was observed by Gloess et al. [44] and Andueza et al. [45]. Furthermore, Gloess et al. [44] found no correlation between the pH and the titratable acidity of the coffee brews. The authors explain that many of the acids presented in the coffee infusion may not be completely deprotonated at pH measurement of this infusions and as a consequence does not affect its pH, but could be measured during titration with alkali.

The evaluation of the impact of water temperature in the hot brew method on the pH showed that infusions prepared with water at 95 °C were often characterized by a more alkaline reaction. Salamanca et al. [46] evaluated the effect of the type of coffee (natural and washed Arabica as well as natural Robusta) and the extraction temperature profile (88–93 °C, 90 °C, 93–88 °C) on the pH of the infusion. The pH varied depending on the type of coffee, as well as the temperature of brewing. In the case of Arabica beans, the infusions prepared at 93–88 °C were more acidic, while those obtained in a constant temperature of 90 °C were more alkaline. In the case of the Robusta variety, the most acidic infusions were those brewed at 88–93 °C, while the alkaline were prepared at 93–88 °C. Regardless of the water temperature, as in our study, all prepared infusions were acidic, and pH ranging from 5.01 ± 0.82 to 5.74 ± 0.04.

The analysis of the influence of the brewing time on the pH of the obtained infusions showed that in the case of the cold brew method, the extracts obtained during a longer brewing time (24 h) had a higher pH, whereas, in the hot brewing technique, the infusions obtained during a shorter brewing time (4 min) had a more alkaline pH. Fuller and Rao [37] analyzed the pH of infusions prepared using the cold brew method for 400 and 1440 min. They observed more alkaline reactions for extracts prepared during a shorter brewing time.

The comparison of the pH of the infusions depending on the country of origin of the beans has shown that in the case of unroasted beans, the most alkaline are usually the infusions from Rwanda coffee, while the most acidic—the beans grown in Brazil. In the case of roasted beans, the most alkaline pH, regardless of the water temperature and infusion time, was found for infusions of beans imported from India, while acidic for extracts obtained from beans grown in Colombia and Peru. Rao and Fuller [27] also compared the pH of infusions made from roasted beans grown in different countries (Brazil, Ethiopia, Myanmar, Colombia, and Mexico). In the case of the hot-brew method, the infusion made from Brazilian beans was the most alkaline, whereas the most acidic was made from the coffee grown in Ethiopia. In the case of cold brewing, the most alkaline was the infusion of beans grown in Myanmar, and the most acidic, also the extracts of Ethiopian beans. In another study of Fuller and Rao [37], it was noted that pH of infusions depended not only on the hot or cold method, but also on the degree of roasting and grinding the beans. The most acidic reaction was found for medium-coarse coffee, while the most alkaline for dark roast and medium coarse ground coffee (dark-medium).

### 2.5. Statistical Analysis

The statistically significant Pearson correlation coefficients were obtained between methods: DPPH vs. ABTS (r = 0.489; *p* < 0.0001), FRAP vs. F-C (r = 0.314; *p* < 0.02), ABTS vs. flavonoids content (r = 0.270; *p* < 0.04) as well as F-C vs. flavonoids content (r = 0.688; *p* < 0.0001). Correlations between the caffeine content and the results of antioxidant activity as well as the total polyphenols and flavonoids content, were also assessed. Statistically significant correlation coefficients were obtained between the caffeine content and antioxidant activity evaluated with DPPH (r = 0.390; *p* < 0.003) and ABTS (r = 0.453; *p* < 0.001) methods. Moreover, a significant correlation was found for caffeine vs. total polyphenols (r = 0.355; *p* < 0.006) and for caffeine vs. flavonoids content (r = 0.445; *p* < 0.001). The statistical significance of differences between the results obtained for infusions prepared using various brewing parameters was also assessed. The differences between the antioxidant activity of infusions prepared from roasted and unroasted beans were statistically significant (z = 4.871; *p* < 0.0001). Caffeine content in infusions of green and brown beans also differed significantly (z = 4.206; *p* < 0.0001). The above-mentioned differences, between the concentration of polyphenols and flavonoids, were statistically insignificant. The assessment of differences between the coffee brewing temperature (CB vs. 84–86 °C and CB vs. 93–95 °C) showed that in the case of antioxidant activity, the differences were statistically significant between the cold-brew method and 93–95 °C (z = 2.274; *p* < 0.03). The content of polyphenols and flavonoids differed significantly between the cold-brew method and 84–86 °C as well as cold brew method and 93–95 °C (z = 4.436 and z = 3.212, respectively; *p* < 0.01). Furthermore, the caffeine content differed depending on the brewing method (CB vs. 84–86 °C—z = 3.808; *p* < 0.001 as well as CB vs. 93–95 °C—z = 2.688; *p* < 0.01). The differences between the brewing temperatures in the hot brew method (84–86 °C vs. 93–95 °C) were not statistically significant taking into account antioxidant activity, polyphenols and flavonoids content, as well as caffeine concentration in the analyzed infusions. The evaluation of differences between shorter and longer brewing time in individual brewing techniques (9 h vs. 24 h and 4 min vs. 10 min) showed that only the differences between caffeine content in cold brew infusions (9 h vs. 24 h), were statistically significant (z = 1.988; *p* < 0.05). The statistically significance of differences between the antioxidant activity, the content of caffeine, polyphenols, and flavonoids in infusions from beans (both roasted and unroasted) cultivated in different countries was also assessed. In the case of antioxidant activity, the differences were statistically significant between coffees imported from Brazil and Colombia (z = 3.806; *p* < 0.001), Colombia and Peru (z = 3.622; *p* < 0.001), as well as Colombia and Rwanda (z = 4.167; *p* < 0.001). The content of polyphenols and flavonoids differed significantly between infusions obtained from beans cultivated in Brazil vs. Colombia (z = 2.057; *p* < 0.04), Brazil vs. Rwanda (z = 4.171; *p* < 0.001), Colombia vs. Rwanda (z = 2.743; *p* < 0.01) as well as Peru vs. Rwanda (z = 2.600; *p* < 0.01). Caffeine content differed significantly between infusions brewed with coffee beans from Brazil and India, Colombia and India, India and Peru as well as India and Rwanda (z = 3.059; *p* < 0.01).

## 3. Materials and Methods

### 3.1. Chemicals

Acetic acid (99.5%), aluminum chloride hexahydrate, copper(II) chloride dihydrate, disodium hydrogen phosphate dihydrate, 96% ethanol, 36% hydrochloric acid, iron(III) chloride hexahydrate, methanol, phosphoric acid, potassium persulfate, potassium dihydrogen phosphate, potassium hexacyanoferrate(III), sodium acetate anhydrous, sodium carbonate anhydrous, sodium hydroxide, sodium nitrite, and trichloroacetic acid were purchased from Chempur, Poland. Neocuproine was delivered by J&K Scientific, Germany. Folin–Ciocalteu reagent, acetonitrile, iron(II) sulfate heptahydrate, gallic acid were supplied by Merck, Germany, whereas rutin trihydrate by Roth, Germany. ABTS (2,2′-azino-bis(3-ethylbenzothiazoline-6-sulfonic acid), DPPH (2,2-diphenyl-1-picrylhydrazyl), TPTZ (2,4,6-tris(2-pyridyl)-s-triazine), Trolox (6-hydroxy-2,5,7,8-tetramethylchromane-2-carboxylic acid), caffeine were purchased from Sigma-Aldrich, USA. All the chemicals were of analytical grade.

### 3.2. Preparation of Coffee Brews

Unroasted and roasted Arabica coffee beans from Brazil, Colombia, India, Peru, and Rwanda were used to prepare the infusions. The region of coffee from individual countries and the degree of roasting are summarized in Table 5. Coffee beans were ground using electric grinder (CTC Clatronic KSW 3306, Germany), immediately before sample preparation. The roasted beans were ground for 15 s, while unroasted, due to the greater hardness, for 70 s, until the coffee was finely ground. The ground beans were poured over with boiled tap water at different temperatures (hot-brew and cold-brew method) and subjected to different brewing times. The details of the various brewing methods are presented in Table 2. The completed brews were filtered through filter papers. Prepared 5% (*w*/*w*) infusions were filtered through Whatman’s filter papers no. 4. All the extracts were stored at +4 °C until the analysis.

### 3.3. Evaluation of Antioxidant Activity

The antioxidant activity of infusions was evaluated by several in vitro methods. The ability to scavenge free radicals (RSA [%]) was assessed by the DPPH and ABTS methods. Moreover, the ability of samples to reduce ferric and cupric ions was evaluated using FRAP (ferric reducing antioxidant power), PFRAP (potassium ferricyanide reducing power), and CUPRAC (cupric ion reducing antioxidant capacity) methods. The evaluation of antioxidant activity by DPPH, ABTS, and FRAP methods was performed as described by Muzykiewicz et al. [47]. To evaluate ferric reducing capacity of infusions, the FRAP method, as described by Apak et al. [48], and the PFRAP technique (with slight modifications), according to Jayaprakasha et al. [49], were used. The incubation time was reduced to 10 min and absorbance was measured at 734 nm. The spectrophotometric measurements were performed in 1 cm cuvettes using Hitachi U-5100 spectrophotometer (Japan). In DPPH and ABTS methods the activity was expressed as RSA [%], whereas in CUPRAC technique as Trolox equivalents (TEAC)—mg Trolox/g RM (raw material). The reducing power evaluated using FRAP and PFRAP method was presented as FeSO_4_ equivalents—mg FeSO_4_/g RM. Three samples were prepared from each extract and the results are presented as an arithmetic mean ± standard deviation (SD).

### 3.4. Evaluation of Total Polyphenols and Flavonoids Content

The total polyphenols content (Folin–Ciocalteu method) was evaluated as described by Muzykiewicz et al. [47], whereas the flavonoids according to Saeed et al. [50]. The spectrophotometric measurements were performed in 1 cm cuvettes using Hitachi U-5100 spectrophotometer (Japan). The total polyphenols content was expressed as gallic acid (GA) equivalents (GAE)—mg GA/g RM, whereas flavonoids content as rutin equivalents—mg rutin/g RM. Three samples were prepared from each extract and the results are presented as an arithmetic mean ± standard deviation (SD).

### 3.5. Evaluation of Tannins Content and pH of Infusions

Tannins content in infusions was analyzed according to Saeed et al. [50]. The few drops of 0.1% FeCl_3_ were added to the coffee infusion. The appearance of a blue color indicated the presence of tannins in the coffee infusion. The pH of brews was measured using Thermo Electron Orion Benchtop 410A pH meter (USA).

### 3.6. HPLC Analysis

The concentration of caffeine in all coffee infusions was determined by high-performance liquid chromatography (HPLC-UV, Knauer, Germany). The tested compound was separated on a 125 × 4 mm column containing Hyperisil ODS (C_18_), particle size 5 μm. The mobile phase consisted of 0.5 M H_3_PO_4_ (pH 2.5), acetonitrile and MeOH in the ratio 180:20:10 (*v*/*v*/*v*), flow rate was 1 mL/min 20 µL of the analyzed sample was injected on the column. The determinations were carried out at 272 nm. The correlation coefficient of the calibration curve was r = 0.999 (y = 370683x + 32.205, retention time—2.05 min). Each sample was analyzed in triplicate, and the results are presented as arithmetic mean ± standard deviation (SD).

### 3.7. Statistical Analysis

The Pearson’s linear correlation between the antioxidant activity (DPPH, ABTS, FRAP, PFRAP, CUPRAC methods), polyphenols, flavonoids as well as caffeine content was determined. The significance of differences between the results of antioxidant activity, the content of polyphenols, flavonoids, and caffeine, obtained for infusions prepared using various parameters (method, time and temperature of brewing), considering the roasting and origin of beans was determined with Wilcoxon signed rank-test (parameter z). *p* < 0.05 was considered to be statistically significant. All the calculations were done with Statistica 13.3PL Software (StatSoft, Poland).

## 4. Conclusions

The results of the study showed that the selection of beans and brewing methods could have a significant effect on antioxidant activity, polyphenols, flavonoids, and caffeine content, as well as the pH of the infusions prepared from Arabica coffee beans. In general, a higher antioxidant activity and content of the above-mentioned biologically active compounds were obtained in the infusions prepared from roasted beans, as compared to the unroasted coffee beans. The origin of the beans and the brewing technique (hot or cold brew) also influenced the tested properties. Cold-brew infusions were generally characterized by a higher caffeine and total polyphenols (including flavonoids) content in the case of unroasted beans. The hot brewing method led to obtain extracts with higher antioxidant activity and the content of phenolic compounds in the case of roasted beans. In this study, the coffee beans were imported from different countries and were characterized by different degree of roasting, which also had a significant impact on the characteristics of infusions. It seems that factors such as brewing time (9 h vs. 24 h as well as 4 min vs. 10 min) and water temperature (below and above 90 °C) had a less significant impact on the tested properties. All infusions were slightly acidic and contained tannins. The results suggest that origin of coffee beans and brewing parameters seem to be responsible for the tested properties of infusions, therefore of their preparation should be optimized to obtain infusions with the most favorable content of biologically active compounds.

## Figures and Tables

**Table 1:** Health-promoting effect of coffee infusions on the human body.

Area of Impact/Type of Dysfunction	Effect
**The nervous system** **The central nervous system (CNS)** - Alzheimer’s disease - Parkinson’s disease - Depression **Peripheral nervous system** - Sense organs	An adenosine receptor antagonist—caffeine stimulates the CNS, improves concentration and thought processes [16,17,18,19]. Coffee consumption improves the metabolism and uptake of the antiparkinsonian drugs, which reduces the latency to a motor response and reduces the risk of Parkinson’s disease [16,17,18]. Caffeine stimulates the secretion of serotonin and dopamine and, in the proper dose, it can reduce the risk of depression [17]. The soluble and volatile infusion components provide aroma and flavor [17,18].
**The gastrointestinal system**	Caffeine stimulates gastric acid secretion by activating bitter taste receptors, what decreases the pH of the gastric juice. Low pH improves the solubility of weakly alkaline drugs as they form soluble salts [16].
**Obesity and type II diabetes (metabolic disorders)**	Chlorogenic acid and trigonelline significantly reduce blood glucose and insulin levels [17,18,19]. Cafestol increases insulin sensitivity in muscle cells [17,18,19]. Chlorogenic acid intensifies the adipose tissue decomposition, and caffeine inhibits the absorption of fatty acids in the intestinal lumen [17,18,19]. Infusions are characterized by a low calorific value [17].
**The cardiovascular system**	Antioxidants improve the bioavailability of nitric oxide in the vascular system and enhance the vascular endothelium [17,18,19].
**Carcinogenesis**	Components contained in coffee repair and protect DNA against oxidative damage, show anti-inflammatory activity, increase apoptosis of cancer cells, and cause tumor suppression [17,18,19,20].
**Large intestine**	Coffee increases intestinal peristalsis, excretion of bile acids with the feces, and modifies the composition of the intestinal microbiota [19].
**Breast cancer**	Phytohormones in coffee increase the concentration of globulin, which binds sex hormones and reduces the absorption of testosterone and affects the level of luteal estrogens [17,20].
**Ovarian cancer**	Chlorogenic, caffeic acids, and caffeine show antiproliferative properties in ovarian cancer cells. Cytochrome P450 isozymes involved in caffeine metabolism may contribute to the development of ovarian cancer or reduce the risk of its occurrence [17,19].
**Endometrial cancer** **(EC)**	This effect of coffee is associated with the correlation between the prevalence of obesity and EC and with the increased sensitivity of cellular receptors to insulin [19].

**Table 2:** Description of the brewing methods.

Signature	Description of the Brewing Methods
**Cold brew**	
CB, 9 h	The ground grains were poured over with boiled and cooled tap water at 23–25 °C and stored at +4 °C for 9 h.
CB, 24 h	The ground grains were poured over with boiled and cooled tap water at 23–25 °C and stored at +4 °C for 24 h.
**Hot brew**	
86 °C, 4’	The ground grains were poured over with boiled tap water at 84–86 °C and brewing for 4 min.
86 °C, 10’	The ground grains were poured over with boiled tap water at 84–86 °C and brewing for 10 min.
95 °C, 4’	The ground grains were poured over with boiled tap water at 93–95 °C and brewing for 4 min.
95 °C, 10’	The ground grains were poured over with boiled tap water at 93–95 °C and brewing for 10 min.

**Table 3:** The mean (±SD) caffeine content in studied infusions. Details regarding brewing methods are summarized in Table 2.

Brewing Method	mg/100 mL of Coffee Infusion	
	Unroasted Bean Infusion	Roasted Bean Infusion
	Brazil	
CB, 9 h	54.13 ± 1.35	48.87 ± 0.08
CB, 24 h	51.18 ± 0.59	67.72 ± 2.36
86 °C, 4’	34.50 ± 0.52	46.00 ± 0.11
86 °C, 10’	42.68 ± 0.33	47.53 ± 0.18
95 °C, 4’	43.22 ± 0.46	53.07 ± 0.39
95 °C, 10’	29.90 ± 0.32	49.08 ± 0.63
	**Colombia**	
CB, 9 h	62.30 ± 0.55	50.61 ± 0.17
CB, 24 h	60.11 ± 0.17	57.04 ± 0.82
86 °C, 4’	36.02 ± 0.03	52.78 ± 0.57
86 °C, 10’	37.64 ± 0.20	48.65 ± 0.24
95 °C, 4’	34.26 ± 0.12	56.77 ± 0.25
95 °C, 10’	32.15 ± 0.07	45.15 ± 0.28
	**India**	
CB, 9 h	65.53 ± 0.09	66.60 ± 0.36
CB, 24 h	65.61 ± 0.16	78.30 ± 0.34
86 °C, 4’	51.32 ± 0.08	53.38 ± 0.09
86 °C, 10’	61.58 ± 0.27	61.22 ± 0.17
95 °C, 4’	49.78 ± 0.13	62.12 ± 0.72
95 °C, 10’	52.93 ± 0.21	57.80 ± 0.56
	**Peru**	
CB, 9 h	38.97 ± 0.22	59.27 ± 0.20
CB, 24 h	41.88 ± 0.10	59.62 ± 0.15
86 °C, 4’	27.79 ± 0.15	44.73 ± 0.34
86 °C, 10’	27.23 ± 0.07	57.27 ± 0.23
95 °C, 4’	18.15 ± 0.30	53.98 ± 0.20
95 °C, 10’	28.68 ± 0.18	51.80 ± 0.35
	**Rwanda**	
CB, 9 h	39.26 ± 0.14	50.08 ± 0.20
CB, 24 h	49.01 ± 0.45	54.01 ± 0.07
86 °C, 4’	27.80 ± 0.13	49.38 ± 0.14
86 °C, 10’	29.75 ± 0.43	52.77 ± 0.20
95 °C, 4’	25.72 ± 0.22	49.25 ± 0.43
95 °C, 10’	47.32 ± 0.47	50.63 ± 0.30

**Table 4:** pH of coffee infusions. Details regarding brewing methods are summarized in Table 2.

pH of Coffee Infusion					
**infusion of unroasted beans**	**Brazil**	**Colombia**	**India**	**Peru**	**Rwanda**
CB, 9 h	6.04	5.75	6.00	5.17	5.16
CB, 24 h	5.92	6.11	6.24	6.17	6.44
86 °C, 4’	6.11	6.48	6.40	6.47	6.35
86 °C, 10’	6.06	6.28	6.20	5.94	6.50
95 °C, 4’	6.26	6.13	6.26	6.58	6.50
95 °C, 10’	6.16	6.32	6.29	6.23	6.35
**infusion of roasted beans**	**Brazil**	**Colombia**	**India**	**Peru**	**Rwanda**
CB, 9 h	5.25	5.07	5.62	5.12	4.99
CB, 24 h	5.31	5.07	5.71	5.19	5.21
86 °C, 4’	5.15	5.07	5.50	5.05	5.17
86 °C, 10’	5.14	5.03	5.48	5.07	5.14
95 °C, 4’	5.19	5.06	5.53	5.11	5.12
95 °C, 10’	5.20	5.07	5.50	5.02	5.10

**Table 5:** The origin of the analyzed coffee beans.

Country of the Beans Origins	Region	Roasting Degree
Brazil (Cerrado)	Cerrado Mineiro	medium
Colombia (Medellin)	Antioquia/Medellin	medium light
India (Monsooned Malabar)	Karnataka, Western Ghats	medium
Peru (Cepro Yanesha)	Villa Rica, Oxapampa, Pasco	medium dark
Rwanda (Sake)	Ngoma District, Eastern Province	light

## Data Availability

The data presented in this study are available in this article.

## References

[1] Dos Santos A., Marques L., Gonçalves C., Marcucci M. (2019). Botanical aspects, caffeine content and antioxidant activity of Coffea arabica. Am. J. Plant. Sci..

[2] Goodin M., Dos Reis Figueira A. (2019). Good to the last drop: The emergence of coffee ringspot virus. PLoS Pathog..

[3] Matysek-Nawrocka M., Cyrankiewicz P. (2016). Biological active substances derived from tea, coffee and cocoa and their application in cosmetics. Post. Fitoter..

[4] Krishnan S. (2017). Sustainable coffee production. ORE Environ. Sci..

[5] Kohlmünzer S. (2013). Pharmacognosy.

[6] Murata M. (2021). Browning and pigmentation in food through the Maillard reaction. Glycoconj J..

[7] Janda K., Jakubczyk K., Baranowska-Bosiacka I., Kapczuk P., Kochman J., Rebacz-Maron E., Gutowska I. (2020). Mineral composition and antioxidant potential of coffee beverages depending on the brewing method. Foods.

[8] Cordoba N., Fernandez-Alduenda M., Moreno F.L., Ruiz Y. (2020). Coffee extraction: A review of parameters and their influence on the physicochemical characteristics and flavour of coffee brews. Trends Food Sci. Technol..

[9] Kim S.Y., Kang B.S. (2018). A colorimetric sensor array-based classification of coffees. Sens. Actuators B Chem..

[10] Gonzales E.C.I., Lloren K.G.M., Al-shdifat J.S., Valdez L.B., Gines K.R., Garcia E.V. (2018). Effect of pressure on the particle size distribution of espresso coffee. KIMIKA.

[11] Cordoba N., Pataquiva L., Osorio C., Leonardo Moreno F., Yolanda Ruiz R. (2019). Effect of grinding, extraction time and type of coffee on the physicochemical and flavour characteristics of cold brew coffee. Sci. Rep..

[12] Angeloni G., Guerrini L., Masella P., Innocenti M., Bellumori M., Parenti A. (2018). Characterization and comparison of cold brew and cold drip coffee extraction methods. J. Sci. Food Agric..

[13] Amanpour A., Selli S. (2016). Differentiation of volatile profiles and odor activity values of Turkish coffee and French press coffee. J. Food Process. Preserv..

[14] Mubarak A., Croft K.D., Bondonno C.B., Din N.S. (2019). Comparison of liberica and arabica coffee: Chlorogenic acid, caffeine, total phenolic and DPPH radical scavenging activity. Asian J. Agric. Biol..

[15] Affonso R., Voytena A., Fanan S., Pitz H., Coelho D., Horstmann A., Pereira A., Uarrota V., Hellmann M., Ravela L. (2016). Phytochemical composition, antioxidant activity, and the effect of the aqueous extract of Coffee (*Coffea arabica* L.) Bean residual press cake on the skin wound healing. Oxid. Med. Cell Longev..

[16] Belayneh A., Molla F. (2020). The Effect of Coffee on Pharmacokinetic Properties of Drugs: A Review. BioMed. Res. Int..

[17] Pelczyńska M., Bogdański P. (2019). Health-promoting properties of coffee. Varia Med..

[18] Gemechu F.G. (2020). Embracing nutritional qualities, biological activities and technological properties of coffee by products in functional food formulation. Trends Food Sci. Technol..

[19] Witkowska A., Mirończuk-Chodakowska I., Terlikowska K., Kulesza K., Zujko M. (2020). Coffee and its biologically active components: Is there a connection to breast, endometrial, and ovarian cancer?—A review. Pol. J. Food Nutr. Sci..

[20] Aguiar J., Estevinho B.N., Santos L. (2016). Microencapsulation of natural antioxidants for food application—The specific case of coffee antioxidants—A review. Trends Food Sci. Technol..

[21] Priftis A., Stagos D., Konstantinopoulos K., Tsitsimpikou C., Spandidos D.A., Tsatsakis A.M., Tzatzarakis M.N., Kouretas D. (2015). Comparison of antioxidant activity between green and roasted coffee beans using molecular methods. Mol. Med. Rep..

[22] Dybkowska E., Sadowska A., Rakowska R., Dębowska M., Świderski F., Świąder K. (2017). Assessing polyphenols content and antioxidant activity in coffee beans according to origin and the degree of roasting. Rocz. Panstw. Zakl. Hig..

[23] Ribeiro E., de Souza Rocha T., Prudencio S.H. (2021). Potential of green and roasted coffee beans and spent coffee grounds to provide bioactive peptides. Food Chem..

[24] Hečimović I., Belščak-Cvitanović A., Horžić D., Komes D. (2011). Comparative study of polyphenols and caffeine in different coffee varieties affected by the degree of roasting. Food Chem..

[25] Oszmiański J., Lachowicz S., Gławdel E., Cebulak T., Ochmian I. (2018). Determination of phytochemical composition and antioxidant capacity of 22 old apple cultivars grown in Poland. Eur. Food Res. Technol..

[26] Oszmiański J., Lachowicz S., Gamsjäger H. (2020). Phytochemical analysis by liquid chromatography of ten old apple varieties grown in Austria and their antioxidative activity. Eur. Food Res. Technol..

[27] Rao N.Z., Fuller M. (2018). Acidity and antioxidant activity of cold brew coffee. Sci. Rep..

[28] Rao N.Z., Fuller M., Grim M.D. (2020). Physiochemical characteristics of hot and cold brew coffee chemistry: The effects of roast level and brewing temperature on compound extraction. Foods.

[29] Castiglioni S., Damiani E., Astolfi P., Carloni P. (2015). Influence of steeping conditions (time, temperature, and particle size) on antioxidant properties and sensory attributes of some white and green teas. Int. J. Food Sci. Nutr..

[30] Pramudya R.C., Seo H.S. (2018). Influences of product temperature on emotional responses to, and sensory attributes of, coffee and green tea beverages. Front. Psychol..

[31] Górecki M., Hallmann E. (2020). The antioxidant content of coffee and its in vitro activity as an effect of its production method and roasting and brewing time. Antioxidants.

[32] Król K., Gantner M., Tatarak A., Hallmann E. (2020). The content of polyphenols in coffee beans as roasting, origin and storage effect. Eur. Food Res. Technol..

[33] Fibrianto K., Umam K., Wulandari E.S. (2018). Effect of roasting profiles and brewing methods on the characteristics of bali kintamani coffee. Adv. Eng. Res..

[34] Merecz A., Marusińska A., Karwowski B.T. (2018). The content of biologically active substances and antioxidant activity in coffee depending on brewing method. Pol. J. Natur. Sci..

[35] Motora K.G., Beyene T.T. (2017). Determination of caffeine in raw and roasted coffee beans of ilu abba bora zone, South West Ethiopia. Indo Am. J. Pharm. Res..

[36] Gebeyehu B.T., Bikila S.L. (2015). Determination of caffeine content and antioxidant activity of coffee. Am. J. Appl. Chem..

[37] Fuller M., Rao N.Z. (2017). The effect of time, roasting temperature, and grind size on caffeine and chlorogenic acid concentrations in cold brew coffee. Sci. Rep..

[38] Cόrdoba N., Moreno F.L., Coralia O., Velaśqueaz S., Ruiz Y. (2021). Chemical and sensory evaluation of cold brew coffees using different roasting profiles and brewing methods. Food Res. Int..

[39] Caprioli G., Cortese M., Maggi F., Minnetti C., Odello L., Sagratini G., Vittori S. (2014). Quantification of caffeine, trigonelline and nicotinic acid in espresso coffee: The influence of espresso machines and coffee cultivars. Int. J. Food Sci. Nutr..

[40] Caprioli G., Cortese M., Sagratini G., Vittori S. (2015). The influence of different types of preparation (espresso and brew) on coffee aroma and main bioactive constituents. Int. J. Food Sci. Nutr..

[41] Zaguła G., Bajcar M., Saletnik B., Czernicka M., Puchalski C., Kapusta I., Oszmiański J. (2017). Comparison of the effectiveness of water-based extraction of substances from dry tea leaves with the use of magnetic field assisted extraction techniques. Molecules.

[42] Choi B., Koh E. (2017). Spent coffee as a rich source of antioxidative compounds. Food Sci. Biotechnol..

[43] Patay É.B., Sali N., Kőszegi T., Csepregi R., Balázs V.L., Németh T.S., Németh T., Papp N. (2016). Antioxidant potential, tannin and polyphenol contents of seed and pericarp of three Coffea species. Asian Pac. J. Trop Med..

[44] Gloess A.N., Schönbächler B., Klopprogge B., D’Ambrosio L., Chatelain K., Bongartz A., Strittmatter A., Rast M., Yeretzian C. (2013). Comparison of nine common coffee extraction methods: Instrumental and sensory analysis. Eur. Food Res. Technol..

[45] Andueza S., Vila M.A., Paz de Peña M., Cid C. (2007). Influence of coffee/water ratio on the final quality of espresso coffee. J. Sci. Food Agric..

[46] Salamanca C.A., Fiol N., González C., Saez M., Villaescusa I. (2017). Extraction of espresso coffee by using gradient of temperature. Effect on physicochemical and sensorial characteristics of espresso. Food Chem..

[47] Muzykiewicz A., Zielonka-Brzezicka J., Klimowicz A. (2019). The antioxidant potential of flesh, albedo and flavedo extracts from different varieties of grapefruits. Acta Sci. Pol. Technol. Aliment..

[48] Apak R., Güçlü K., Özyürek M., Karademir S.E. (2004). Novel total antioxidant capacity index for dietary polyphenols and vitamins C and E, using their cupric ion reducing capability in the presence of neocuproine: CUPRAC method. J. Agric. Food Chem..

[49] Jayaprakasha G.K., Singh R.P., Sakariah K.K. (2001). Antioxidant activity of grape seed (*Vitis vinifera*) extracts on peroxidation models in vitro. Food Chem..

[50] Saeed N., Khan M.R., Shabbir M. (2012). Antioxidant activity, total phenolic and total flavonoid contents of whole plant extracts *Torilis leptophylla* L.. BMC Complement. Altern. Med..

